# Exploring Early Detection of Frailty Syndrome in Older Adults: Evaluation of Oxi-Immune Markers, Clinical Parameters and Modifiable Risk Factors

**DOI:** 10.3390/antiox10121975

**Published:** 2021-12-10

**Authors:** Armanda Teixeira-Gomes, Blanca Laffon, Vanessa Valdiglesias, Johanna M. Gostner, Thomas Felder, Carla Costa, Joana Madureira, Dietmar Fuchs, João Paulo Teixeira, Solange Costa

**Affiliations:** 1 EPIUnit—Instituto de Saúde Pública, Universidade do Porto, Rua das Taipas 135, 4050-600 Porto, Portugal; armandatgomes@gmail.com (A.T.-G.); carla.trindade@insa.min-saude.pt (C.C.); jvmadureira@gmail.com (J.M.); solange.costa2@gmail.com (S.C.); 2 Environmental Health Department, National Institute of Health Doutor Ricardo Jorge, Rua Alexandre Herculano 321, 4000-055 Porto, Portugal; 3 School of Medicine and Biomedical Sciences (ICBAS), University of Porto, Rua de Jorge Viterbo Ferreira 228, 4050-313 Porto, Portugal; 4 Laboratory for Integrative and Translational Research in Population Health (ITR), Rua das Taipas 135, 4050-600 Porto, Portugal; 5 Centro de Investigaciones Científicas Avanzadas (CICA), Grupo DICOMOSA, Departamento de Psicología, Facultad de Ciencias de la Educación, Campus Elviña s/n, Universidade da Coruña, 15071 A Coruña, Spain; blaffon@udc.es; 6 Instituto de Investigación Biomédica de A Coruña (INIBIC), AE CICA-INIBIC. Oza, 15071 A Coruña, Spain; vvaldiglesias@udc.es; 7 Centro de Investigaciones Científicas Avanzadas (CICA), Grupo NanoToxGen, Departamento de Biología, Facultad de Ciencias, Campus A Zapateira s/n, Universidade da Coruña, 15071 A Coruña, Spain; 8 Institute of Medical Biochemistry, Biocenter, Medical University of Innsbruck, 6020 Innsbruck, Austria; johanna.gostner@i-med.ac.at; 9 Department of Laboratory Medicine, Paracelsus Medical University, 5020 Salzburg, Austria; t.felder@salk.at; 10 Institute of Biological Chemistry, Biocenter, Medical University of Innsbruck, 6020 Innsbruck, Austria; dietmar.fuchs@i-med.ac.at

**Keywords:** frailty, oxi-immune markers, CRP and IL-6, Kyn/Trp, neopterin, Phe/Tyr, nitrite, oxidative DNA damage, vitamin A and vitamin E, modifiable risk factors

## Abstract

Ageing is accompanied with a decline in several physiological systems. Frailty is an age-related syndrome correlated to the loss of homeostasis and increased vulnerability to stressors, which is associated with increase in the risk of disability, comorbidity, hospitalisation, and death in older adults. The aim of this study was to understand the relationship between frailty syndrome, immune activation, and oxidative stress. Serum concentrations of vitamins A and E were also evaluated, as well as inflammatory biomarkers (CRP and IL-6) and oxidative DNA levels. A group of Portuguese older adults (≥65 years old) was engaged in this study and classified according to Fried’s frailty phenotype. Significant increases in the inflammatory mediators (CRP and IL-6), neopterin levels, kynurenine to tryptophan ratio (Kyn/Trp), and phenylalanine to tyrosine ratio (Phe/Tyr), and significant decreases in Trp and Tyr concentrations were observed in the presence of frailty. IL-6, neopterin, and Kyn/Trp showed potential as predictable biomarkers of frailty syndrome. Several clinical parameters such as nutrition, dependency scales, and polypharmacy were related to frailty and, consequently, may influence the associations observed. Results obtained show a progressive immune activation and production of pro-inflammatory molecules in the presence of frailty, agreeing with the inflammageing model. Future research should include different dimensions of frailty, including psychological, social, biological, and environmental factors.

## 1. Introduction

People all around the world are living longer. According to the World Health Organization, the population aged 60 years old and over is estimated to increase from 1 billion in 2019 to 2.1 billion by 2050 [1]. This demographic shift leads to several challenges and opportunities and the need for adaptation in our societies. The heterogeneity of human ageing steered researchers and clinicians to pursue a better understanding of the basis of healthy ageing resulting in an increased interest in studying the frailty syndrome [2]. The early identification of people at risk of frailty will allow implementing preventive actions and specialised care, improving the quality of life in old age, and reducing healthcare costs.

Frailty is a recognised geriatric syndrome characterised by increased vulnerability to internal and external stressors, increasing the risk of poor health outcomes such as disability, hospitalisation, institutionalisation, and ultimately death in older adults [3]. As a multidimensional syndrome, frailty roots are complex and usually established on the interaction of genetic, biological, physical, psychological, social, and environmental factors [4]. Given the complex basis of frailty, different models for identification of frailty syndrome emerged, without international agreement [5]. The most popular are the Frailty Index developed by Rockwood et al. [6] and the Frailty Phenotype developed by Fried et al. [7]. Even though Rockwood’s Frailty Index is more comprehensive than the Fried’s Frailty model, since it includes a series of ‘deficits’ (symptoms, signs, diseases, and disabilities), its practical application is difficult in both clinical and research settings due to the extensive number of variables considered [8,9]. Thus, the identification of frailty based on five phenotypical criteria defined by Fried et al. [7] is the most commonly used tool by clinicians and researchers [10].

While developing and validating frailty as a geriatric syndrome, researchers had the need to differentiate the clinical manifestation of frailty from other age-related changes/diseases [7]. As previously reported herein, Fried el at. [7] developed a clinical tool in this regard excluding potentially confounding disease and showing associations between frailty and other pathophysiologies [11,12,13,14]. Nevertheless, the relevance of the biological basis of frailty was not addressed at the same pace. Studies on the underlying biology of frailty were later driven considering the homeostatic and mechanistic pathways previously addressed in ageing research [15].

The ageing process has been associated with the progressive impairment of the immune system response resulting in a low-grade chronic pro-inflammatory status, called “inflammageing” [16] and in a decrease of the adaptative immune response defined as “immunosenescence” [17]. An “oxi-inflamm-aging” integrative model was proposed integrating oxidation and inflammation [18] to explain the deterioration of homeostasis and impairment of physiological systems with advancing age.

Strong evidence suggests that inflammageing is a risk factor for several age-related diseases such as cardiovascular disease [19], osteoporosis [20], Alzheimer’s disease [21], and type-2 diabetes [22].

Inflammageing is associated with increases in levels of acute phase proteins as C-reactive protein (CRP) and pro-inflammatory cytokines as interleukin 6 (IL-6), challenging immunological homeostasis [23]. In the last decade, several studies have established an association between increases in CRP and IL-6 levels and frailty syndrome in older adults [24,25,26,27].

Furthermore, inflammatory cytokines, most importantly interferon gamma (IFN-γ), are known to induce enzymes involved in immunometabolism such as indoleamine 2,3-dioxygenase 1 (IDO-1) and guanosine triphosphate cyclohydrolase I (GTP-CH-I), preferentially in monocytes/macrophages and dendritic cells [28]. As a result of immune activation, IDO-1 activity increases and enhances the degradation of tryptophan (Trp) into kynurenine (Kyn), resulting in a higher kynurenine to tryptophan ratio (Kyn/Trp). Kyn/Trp, which reflects Trp catabolism, can be used to indicate IDO-1 activity if other markers of immune activation are elevated in parallel [29]. GTP-CH-I is a key enzyme in the biochemical pathway leading to neopterin formation in human monocytes/macrophages, as well as to tetrahydrobiopterin (BH4) formation in other cell types. BH4 is a cofactor required by several monooxygenases, including phenylalanine 4-hydroxylase (PHA), involved in the conversion of phenylalanine (Phe) to tyrosine (Tyr); and nitric oxide synthases (NOS), which catalyse the conversion of arginine to nitric oxide (NO), and tryptophan 5-hydroxylase, which is involved in the first step of the pathway that synthesizes serotonin from Trp. BH4 is an oxidation labile molecule, thus a prooxidative milieu present during periods of immune activation affects the activity of these enzymes [30]. Phenylalanine to tyrosine ratio (Phe/Tyr) can be considered as an estimate of PHA activity under proinflammatory conditions [31]. Studies show an association between augmented Trp breakdown and increased neopterin synthesis in older adults [32] and, importantly, that frailty status in aged populations is related to alterations in IDO-1 and GTP-CH-I dependent enzymatic pathways [31,33,34]. Marcos-Pérez et al. [33] found significant increases in neopterin levels, Kyn/Trp, and Phe/Tyr, and decreases in Trp, nitrite (used as surrogate of NO production) and Tyr concentrations in frail individuals compared with non-frail. A study with 73 older adults from South Korea also reported a marked increase in Kyn levels with frailty [34]. Furthermore, lifestyle and nutrition are well known to affect these immunobiochemical circuits [30].

One main evidence for damage to tissues and cells relies on the chronic damage that results from oxidative stress promoted on the organism by free radicals. Reactive oxygen species (ROS) are generated as normal cellular metabolism products, although they can induce undesired damaging and oxidising effects to molecules such as proteins, DNA, and lipids. As previously mentioned, some authors support the theory that oxidative and inflammatory stress are the basis of age-related changes in immune function—“oxi-inflamm-aging” [18]. In fact, the role of oxidative stress in age and age-related diseases is well recognised [35]. Several endpoints have been suggested as potential oxidative stress biomarkers in frailty syndrome: ROS [36,37], oxidised to reduced glutathione ratio [38], protein carbonyls [38], 8-hydroxy-2′-deoxyguanosine (8-OHdG) [39], total antioxidant potential [36], and vitamin E levels [40]. Nevertheless, the referred alterations are not consistent through studies [40,41].

The adverse effects promoted by oxidative damaging species are usually balanced by antioxidants; however, as ageing increases there is an imbalance between levels of antioxidants and production of damaging species. Vitamins such as vitamin A and E are often referred to as “antioxidant vitamins”. They have been suggested as limiting factors to oxidative damage in humans, decreasing the risk of some chronic diseases [42].

The aim of the present study was to delve into the relationship between frailty syndrome and immune system and oxidative stress, through the analysis of several biomarkers involved in IDO-1 and GTP-CH-I pathways and PHA activities. Serum concentrations of vitamins A and E were also evaluated, as well as inflammatory biomarkers (CRP and IL-6) and oxidative DNA levels.

## 2. Materials and Methods

### 2.1. Study Population

In total, 291 individuals (121 males and 170 females) were enrolled in the study. Older adults aged 65 years old or over were recruited from community-dwelling and care centres (nursing homes and day care centres) in the metropolitan region of Porto, Portugal. All participants were informed of the goals of the study, nature of participation risks and benefits, and asked to sign an informed consent form prior to be included in the study. Exclusion criteria included severe dementia and/or cognitive impairment, lack of ability to communicate, severe impairment of sight and hearing, and receiving palliative care. A comprehensive geriatric evaluation was carried out comprising socio-clinical information (medical history, lifestyle, and demographic data), including nutrition screening assessed by the Mini Nutritional Assessment Short-Form (MNA-SF) [43]. Similarly, standard geriatric scales were used to evaluate functional status of the individuals, such as the basic activities of daily living (BADL) by Katz Index [44], and instrumental activities of daily living (IADL) by Lawton-Brody scale [45].

The study was approved by the Ethics Committee of the Instituto de Saúde Pública da Universidade do Porto (ISPUP) (No. CE17081) and authorised by the National Committee for Data Protection (CNPD-Comissão Nacional de Proteção de Dados) (Authorisation No. 5446/2018).

### 2.2. Frailty Status Evaluation

Frailty status was determined according to the five phenotypic criteria proposed by Fried et al. [7], with minor modifications [46]. These criteria are based on the presence or absence of the following parameters: (i) unintentional weight loss (self-reported), (ii) exhaustion (self-reported), (iii) low physical activity (adapted Portuguese version of the Spanish Minnesota Leisure Time Activities short version questionnaire (VREM) [47], converting weekly tasks to equivalent kilocalories of expenditure), (iv) slow walking speed, and (v) muscle weakness (grip strength measured by a JAMAR^®^ Hydraulic Hand Dynamometer). Each criterion was punctuated with “zero” if absent or with “one” if present, following the cut-offs established by Fried’s model [7]. Participants scoring “one” for three or more criteria were classified as frail; one or two criteria, pre-frail; no criteria, non-frail. Anthropometric measures were assessed for cut-off application: weight was measured using a SECA^®^ 761 flat mechanic scale to the nearest 0.5 kg (SECA GMBH & Co. Kg., Hamburg, Germany) and height recorded to the nearest millimetre using a portable stadiometer SECA^®^ 213 (SECA GMBH & Co. Kg., Hamburg, Germany).

### 2.3. Biological Sample Collection

Whole blood samples were obtained by venepuncture and collected into vacutainer tubes containing potassium heparin for plasma, no anticoagulant for serum and BD Vacutainer^®^ CPT™ tubes with sodium citrate for peripheral blood mononuclear cells, early in the morning to avoid circadian variations. Samples were transported in a cooler (4 °C, transport within 30 min maximum), and immediately processed for the different biological endpoints. After centrifugation at 769× *g* for 10 min the serum (following 1–2 h rest at room temperature) and plasma were aliquoted and stored at −80 °C until analysis of immune and inflammatory biomarkers. Peripheral blood mononuclear cells were isolated from BD Vacutainer^®^ CPT™ tubes with sodium citrate (Becton, Dickinson and Company) for oxidative DNA damage evaluation. All samples were coded and analysed under blinded conditions.

### 2.4. CRP Test and IL-6 Immunoassay

Serum levels of CRP were measured by a particle-enhanced immunoturbidimetry method (CRPLX, catalog no. 20764970; Roche Diagnostics, Basel, Switzerland) in a Cobas Integra 400 analyser (Roche Diagnostics) according to manufacturer’s instructions with a detection limit of 0.06 mg/L. Quantification of serum IL-6 levels was performed using a Human IL-6 commercial Kit through the Quantikine enzyme linked immuno-sorbent assay (ELISA) (R&D Systems, Minneapolis, MN, USA) following manufacturer’s standard protocol with an extended quantification range of 3.1–300 pg/mL.

### 2.5. Neopterin, Tryptophan, Kynurenine, Phenylalanine, Tyrosine and Nitrite Analyses

Serum neopterin concentrations were obtained using an ELISA (BRAHMS GmbH, Hennigsdorf, Germany) with a sensitivity of 2 nmol/L neopterin, according to manufacturer’s instructions.

Trp, Kyn, Phe, and Tyr levels were determined by high-performance liquid chromatography (HPLC), as described by Laich et al. [48] and Neurauter et al. [49]. The Trp breakdown, as an indicator of IDO-1 activity (if other immune activation markers are elevated in parallel [50]) was obtained by calculating the Kyn/Trp, and the Phe/Tyr ratio was used as a surrogate of PHA activity [49]. In brief, sample preparation consisted of protein precipitation with trichloroacetic acid and addition of 3-nitro-l-tyrosine (NT) as internal standard. Chromatographic separation was achieved using a LiChroCART RP-18 endcapped column (55-4, 3 µm, Merck, Darmstadt, Germany) together with a reversed-phase C18 pre-column and 15 mmol/L acetic acid-sodium acetate solution (pH = 4.0) (for Trp and Kyn) or 15 mM potassium dihydrogen-phosphate (for Phe and Tyr) as mobile phase. Trp, Phe and Tyr detection was based on their native fluorescence (Trp: excitation (Ex) wavelength (λ) 286 nm, emission (Em) (λ) 366 nm; Phe, Tyr: λ Ex 210 nm, λ Em 302 nm). UV-detection at 360 nm wavelength was used for Kyn and NT. Method validation included linearity (*R*^2^ ≥ 0.998, all analytes), interday precision (Trp: ≤ 1.8%; Kyn: ≤ 4.2%; Phe ≤ 10.0%; Tyr ≤ 5.1%) and recovery (Trp: 99.3%; Kyn: 102.3; Phe: 98.2%; Tyr: 98.8%) [48,49]. Limits of detection were 0.1 µmol/L for Trp, 0.5 µmol/L for Kyn, and 0.3 µmol/L for Phe and Tyr.

As a surrogate marker of nitric oxide (NO) production, the stable NO metabolite nitrite was measured using a modified Griess assay (Merck KGaA, Darmstadt, Germany) with a detection limit of 1.5 µmol/L nitrite [51].

### 2.6. Vitamin A and E Determinations

Quantification of vitamin A and E was performed with the CE-IVD validated assay (Chromsystems, Munich, Germany, order number: 34,000), according to the manufacturer’s instructions on a Nexera XR CL HPLC-PDA system (Shimadzu, Kyoto, Japan) at 325 nm and 295 nm, respectively. The lower limit of quantification was 0.02 mg/L for vitamin A and 0.25 mg/L for vitamin E, respectively.

### 2.7. Enzyme-Modified Comet Assay

Oxidative DNA damage was evaluated using the medium-throughput comet assay using 12-Gel Comet Assay Unit™ (Severn Biotech Ltd., Worcestershire, United Kingdom) following Teixeira-Gomes et al. 2020 [46] and others [52,53]. Cells were suspended in 0.6% (*w*/*v*) low-melting point agarose (Sigma-Aldrich, St. Louis, MO, USA) and 5 μL were dropped onto a frosted slide pre-coated with 1% normal melting point agarose (Lonza, Basel, Switzerland). After 60 min in lysis solution (2.5M NaCl, 100 mM Na2EDTA, 10 mM Tris-base, 0.25M NaOH, pH 10; 1% Triton X100) in the dark, at 4 °C, slides were washed three times (5 min each) and then lymphocytes were incubated with buffer F alone or with Formamidopyrimidine DNA glycosylase (FPG) (8000 units/mL, acquired from New England Biolabs^®^ (Ipswich, MA, USA) and used according to manufacturers’ instructions), for 30 min at 37 °C. Subsequently, slides were immersed in cold alkaline electrophoresis solution (1 mM Na_2_EDTA, 300 mM NaOH, pH 13) for 30 min in the dark, for DNA unwinding. Electrophoresis followed for 20 min at approximately 1 V/cm. Mini-gels were neutralised with cold PBS (10 min) and washed with distilled H_2_O (10 min), dehydrated and fixed with ethanol 70% (15 min) and 96% (15 min) and let to dry overnight. Dried slides were stained with SYBR™ Gold (Invitrogen™, Waltham, MA, USA) at the dilution recommended by the manufacturer at room temperature for 30 min. Microscopic analyses were performed blindly by the same reader on a Nikon Eclipse E400 Epi-fluorescence microscope (G2A filter, Nikon C-SH61, Tokyo, Japan). Comet Assay IV (Perceptive Instruments, Cambridge, United Kingdom) was the semi-automated system used for image capture and analysis. A total of 150 cells were scored for each subject and the percentage of DNA in the comet tail (%tDNA) chosen to evaluate the DNA damage at cell level. Net FPG-sensitive sites were calculated by subtracting the %tDNA values obtained from buffer and enzyme-incubation slides.

### 2.8. Statistical Analysis

A general description of the study population according to frailty status was performed through univariate analysis. The distribution in the three study groups comparing sociodemographic, clinical characteristics, and lifestyle factors was evaluated using analysis of variance (ANOVA) for continuous variables and Chi-square test for categorical variables.

Preliminary univariate analysis of the studied biomarkers according to frailty status was assessed by ANOVA and Tukey’s post-hoc test. Data on CRP, Trp, Tyr, Phe, Phe/Tyr and vitamin E levels followed a normal distribution (Kolmogorov-Smirnov goodness-of-fit test). The remaining endpoints were transformed to achieve a better approximation to the normal distribution. A log-transformation was applied to Kyn, Kyn/Trp, nitrite, vitamin A, net FPG-sensitive sites (oxidative DNA damage), and IL-6, whereas a reciprocal transformation was applied to neopterin.

Linear regression analysis on the log-transformed data was used to estimate the effect of frailty status on the studied parameters. All models were adjusted for age, gender, smoking habits (non-smokers/ever smokers), and actual confounders.

Possible associations between endpoints were calculated by correlation coefficients (Spearman’s rho). To assess biomarker’s ability for frailty syndrome predictivity in our study population, receiver-operating characteristic (ROC) curves were computed. The level of statistical significance was set at 0.05. All analyses were performed using the STATA/SE software package V. 12.0 (StataCorp LP, College Station, TX, USA) and the IBM SPSS software package V. 20 (IBM Corp., Armonk, NY, USA).

## 3. Results

### 3.1. Study Population

The characteristics of the study population according to the phenotypic frailty status are listed in Table 1. Of the total 291 older adults, 111 (38%) were scored as non-frail, 130 (45%) as pre-frail, and 50 (17%) as frail. Study individuals’ age ranged from 65 to 94 years old and significantly increased from non-frail to frail participants. Females were significantly more prevalent than males in pre-frail and frail groups. BMI and smoking habits were consistently distributed among groups. Alcohol consumers were significantly more prevalent in the non-frail group. Regarding polypharmacy, the number of medications taken per day was significantly increased from non-frail < pre-frail < frail subjects. Significant differences were found among groups regarding nutrition, 52% of frail individuals were at risk of malnutrition or malnourished, while 99% of the non-frail subjects had normal nutritional status. As for the functional status scales, 74% of frail individuals were dependent in BADL (e.g., bathing, dressing, toileting) and 90% in IADL (e.g., using the phone, shopping, handling medication), while values in the non-frail group were 11.7% and 8.1%, respectively. No significant differences were observed for vitamin supplementation, vaccination in the last year, number of falls, and self-perceived sight status (data not shown). For self-perceived hearing status, frail subjects reported hearing worse (“Bad”) compared to the other groups (data not shown).

### 3.2. Analysis of the Frailty Phenotype vs. Biomarkers Studied

Data obtained for biological endpoints in the three frailty-phenotype groups are shown in Table 2. Regarding the biomarkers of inflammation, both endpoints, CRP and IL-6, were significantly increased in the presence of frailty. Levels of CRP were significantly increased in pre-frail and frail subjects compared to non-frail (non-frail < pre-frail and frail), while IL-6 concentrations were significantly different in the 3 groups (non-frail < pre-frail < frail). Furthermore, the immune stimulation parameters neopterin, Kyn, Kyn/Trp, and Phe/Tyr also increased significantly with frailty severity. Neopterin and Kyn/Trp significantly differed in the 3 groups (non-frail < pre-frail < frail); while Kyn only increased significantly in frail subjects compared to the other groups (frail > pre-frail and non-frail). Phe/Tyr was increased with frailty severity compared to non-frail (pre-frail and frail> non-frail). On the other hand, a significant decrease in frail group was found for Trp and Tyr concentrations when compared to non-frail and pre-frail (non-frail and pre-frail > frail). No differences between frailty groups were observed for nitrite levels. Considering the results for the antioxidant vitamins A and E, a borderline significant decrease was observed for vitamin E levels in non-frail as compared to frail group (non-frail < frail), no differences were found for vitamin A. For oxidative DNA damage endpoint, significant differences were found among the groups. Net FPG-sensitive sites were significantly increased in pre-frail subjects compared to non-frail (non-frail < pre-frail) and frail (pre-frail > frail).

**Figure 1:**
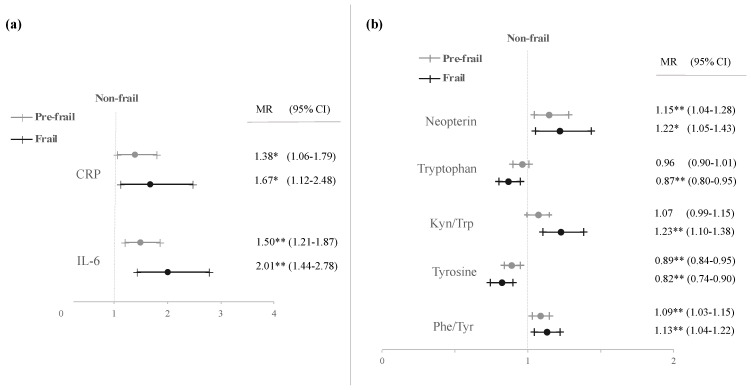
Adjusted mean ratios (MR) with 95% confidence interval (CI) of inflammatory biomarkers (**a**) and immune activation parameters (**b**) in pre-frail and frail subjects. Models adjusted by age, gender, smoking habits, and actual confounders. * *p* < 0.05; ** *p* < 0.01.

A multivariate modelling was carried out to further investigate frailty status adjusted by gender, age, smoking habits, and actual confounders. Data obtained are illustrated in Figure 1 (only significant results). Significant increases with frailty severity in CRP, IL-6, and neopterin levels, and in Kyn/Trp and Phe/Tyr were observed, together with significant decreases in Trp and Tyr concentrations, confirming the results from univariate analysis. Moreover, all significant differences found in the multivariate analysis showed progressive variation with frailty severity, except for Trp and Kyn/Trp, in which the significant differences were only obtained between non-frail and frail subjects.

#### Single Contribution of Each of the 5-Frailty Criteria to the Biomarkers Studied

**Figure 2:**
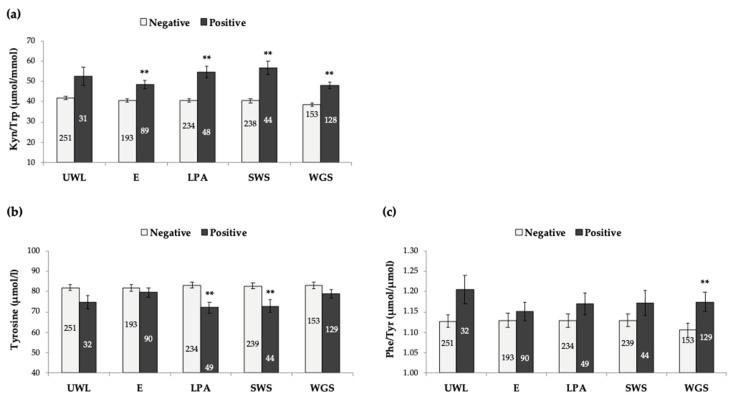
Kyn/Trp (**a**), Tyrosine (**b**) and Phe/Tyr (**c**) in the population analysed, according to each frailty criterion (Fried et al., 2001). Bars represent mean ± standard error. The number of individuals in each group is indicated inside each rod. ** *p* < 0.001, significant difference with regard to negative scoring (Student’s *t*-test). UWL: unintentional weight loss; E: exhaustion; LPA: low physical activity; SWS: slow walking speed; WGS: weakness of grip strength.

Study population was distributed according to their phenotype scoring in each of the 5-frailty criteria defined by Fried’s model (Section 2.2); only biomarkers with significant associations with frailty were analysed (CRP, IL-6, neopterin, Trp, Kyn/Trp, Tyr, and Phe/Tyr). These results can help to further validate Fried’s Frailty phenotype model and can also improve frailty identification by approaching a better combination of specific phenotypic criteria and biomarkers. As illustrated in Figure 2a, Kyn/Trp was increased in older adults scoring positive for exhaustion, low physical activity, slow walking speed and weakness of grip strength, but no association was found with unintentional weight loss. On the other hand, Tyr concentrations were significantly decreased in subjects with low physical activity and slow walking speed (Figure 2b), whereas Phe/Tyr was significantly increased just among older adults showing weakness of grip strength (Figure 2c). For CRP, IL-6, neopterin, and Trp concentrations, significant differences (positive vs. negative) were observed in all 5 frailty criteria (data not shown), so no specific contribution was identified for these biomarkers.

### 3.3. Analysis of Host and Modifiable Lifestyle Factors vs. Biomarkers Studied

The levels of IL-6, neopterin, Kyn, Kyn/Trp, and Tyr were significantly increased with age. Whereas for Trp and Phe/Tyr, a significant decrease was observed (data not shown). For males, a significant increase in nitrite levels was found with regard to females, but the opposite was observed for Tyr, Phe, and vitamin E levels. Regarding smoking habits, increases in IL-6 and neopterin were observed for ever smokers when related to never smokers.

Influence of diet parameters (consumption of dairy products/day, eggs/leguminous > 2/week, meat/fish/poultry every day, fruit/vegetables > 2/day) on the endpoints studied was also explored. A significant increase was observed in Tyr levels for participants that reported to consume at least 2 portions of fruit or vegetables per day, and the inverse correlation was observed for oxidative DNA damage (Table 3).

**Figure 3:**
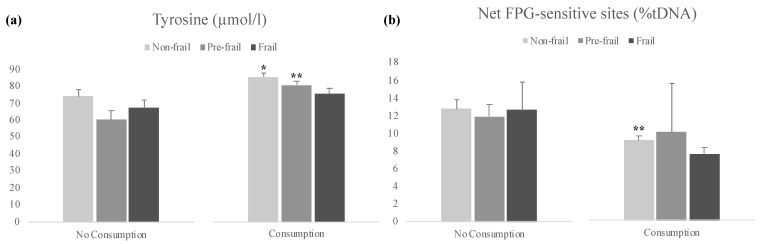
Results of Tyrosine (**a**) and Net FPG-sensitive sites (**b**) by study groups, classified according to fruit/vegetables consumption and frailty status. Bars represent mean ± standard error. * *p* < 0.05, ** *p* < 0.01, significant difference with regard to no consumption within the same frailty group (Student’s *t*-test).

We further investigated the influence of fruit or vegetables consumption and frailty on the aforementioned endpoints, and a significant increase in Tyr levels was observed within non-frail and pre-frail groups when comparing consumers of fruit and vegetables with non-consumers (Figure 3a). Oxidative DNA damage decreased in participants that reported consumption of at least 2 portions of fruit or vegetables per day, significantly in the non-frail group (Figure 3b).

### 3.4. Correlations between the Different Biomarkers Studied

**Figure 4:**
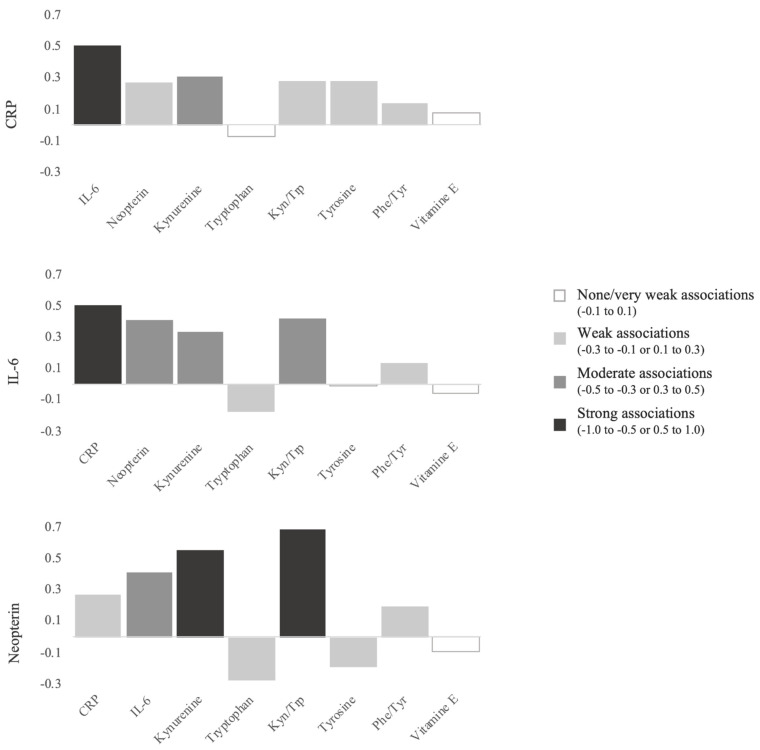
Correlation coefficients between analysed biomarkers.

Figure 4 illustrates the most relevant correlations observed. Important significant positive correlations were found between CRP and IL-6 (*r* = 0.505, *p* < 0.001); CRP and neopterin (*r* = 0.270, *p* < 0.001); CRP and Kyn (*r* = 0.282, *p* < 0.001); CRP and Kyn/Trp (r = 0.282, *p* < 0.001), and CRP and Phe/Tyr (*r* = 0.142, *p* < 0.05). IL-6 was significantly correlated with neopterin (*r* = 0.412, *p* < 0.001), Kyn (*r* = 0.412, *p* < 0.001), and Kyn/Trp (*r* = 0.417, *p* < 0.001). Neopterin also showed positive and strong associations with Kyn (*r* = 0.554, *p* < 0.001) and Kyn/Trp (*r* = 0.687, *p* < 0.001).

### 3.5. Predictive Value of Studied Biomarkers

**Figure 5:**
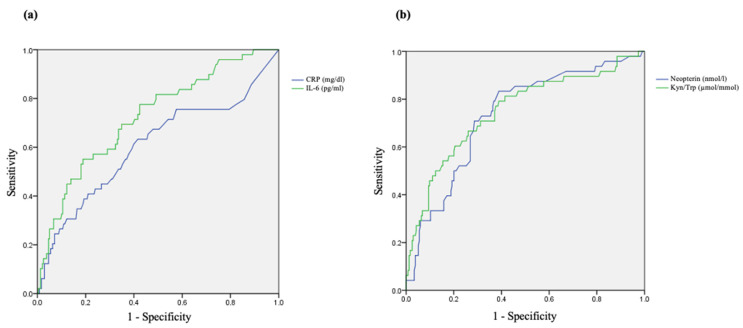
Receiver-operating characteristic (ROC) curves for CRP (**a**), IL-6 (**a**), neopterin (**b**), and Kyn/Trp (**b**) to predict frailty.

ROC curves were computed for biomarkers showing significant association with frailty in our study population, using the non-frail group as the standard. Results obtained are described in Figure 5, only those parameters that presented area under the curve (AUC) values higher than 0.6 are shown. In general, an AUC below 0.6 suggests no discrimination, between 0.6 and 0.7 is satisfactory and 0.7–0.8 is good [54,55]. AUC values found were 0.604 (95% CI 0.51–0.70, *p* < 0.05) for CRP; 0.724 (95% CI 0.65–0.80, *p* < 0.001) for IL-6; 0.733 (95% CI 0.66–0.81, *p* < 0.001) for neopterin, and 0.750 (95% CI 0.67–0.83, *p* < 0.001) for Kyn/Trp. The optimal predictive value for Kyn/Trp was achieved at 44.70 µmol/mmol, with a sensitivity of 66.7% and a specificity of 73.9%. The optimal neopterin concentration obtained was 8.45 nmol/L, with a sensitivity of 83.3%, and a specificity of 61.1%. IL-6 presented an optimal predictive value of 4.45 pg/mL, with a sensitivity of 55.1%, and a specificity of 81.1%. Therefore, for our population the biomarkers with best discriminating ability were Kyn/Trp and neopterin.

## 4. Discussion

Cellular responses to stressors—apoptosis, senescence, and repair—and the systemic response of immune activation/inflammation contribute to the ageing phenotype [56]. To understand the biological basis of frailty, the scientific community hypothesised that the dysregulation of these cellular responses is also likely to contribute to the loss of homeostasis observed in frailty (e.g., cellular senescent phenotype—secreting pro-inflammatory cytokines may contribute to the dysregulated inflammatory state seen in frailty). Researchers also postulated that the patterns observed in different cellular and systemic responses in “normal” ageing could be further accelerated/changed in frailty syndrome [15].

Based on current data associated to the ageing process, the scientific community trusts that some of the biological endpoints related to genomic or immunological alterations might be useful for improving frailty identification in older adults, even before clinical manifestations. Some biological endpoints showed a positive association with frailty syndrome and were suggested as potential biomarkers, but more studies are needed to confirm the results [57,58,59]. The definition of a gold-standard tool for frailty evaluation and larger population studies would lead to more conclusive outcomes promoting scientific consensus [26,60].

So far, to our knowledge, only one pilot study was carried out in the Portuguese population (by our group) assessing the link between biological endpoints and frailty syndrome prevalence in older adults [46]. That study included a smaller population (*n* = 71), and the parameters analysed were essentially environmental factors and genetic biomarkers. Therefore, and given the existing gaps pointed herein, the main goal of the present study was to evaluate frailty syndrome in a group of Portuguese older adults and assess its association with immune stimulation parameters, inflammatory biomarkers, antioxidant vitamins (A and E), and oxidative DNA damage.

The frailty prevalence in our study population, recruited from community settings and care centres, was 38% non-frail, 45% pre-frail, and 17% frail subjects. Our data comply with a recent study reporting a frailty prevalence of 24% non-frail, 54% pre-frail, and 22% frail in a group of 1500 Portuguese older adults (aged 65+) from several regions of the country, living in community-dwelling and retirement homes [61]. In another study also based in Porto area, but comprising only institutionalised subjects, Coelho et al. [62] reported a frailty prevalence of 15% non-frail, 48% pre-frail, and 37% frail, in 252 older adults (aged 65+). Interestingly, in a sample of 338 individuals aged >50 years old, Duarte et al. [63] reported similar proportions of 14% non-frail, 51% pre-frail, and 35% frail subjects in the northern city of Guimarães (no exclusion criteria defined). Both studies are in accordance with the estimated prevalence of frailty syndrome in 250 Spanish older adults (aged 65+), reported in a recent study, 15% non-frail, 50% pre-frail, and 35% frail [64]. Our data from a previously published pilot study comprising 71 community dwelling older adults living in Porto city showed a prevalence of 45% non-frail, 45% pre-frail, and 10% frail [46]. Given the above, it is noticeable that socio-demographic variables of older adults, such as being community dwellers or institutionalised, age, gender, race, may influence frailty prevalence estimates reported in the different studies [65]. Nevertheless, our results are in line with the study comprising the largest population on frailty’s prevalence in Portugal so far, involving all 7 regional areas defined in the Nomenclature of Territorial Units for Statistics (NUTS II) of the country, which confirms the representativity of our study population.

Usually, the prevalence of frailty increases gradually with age, with frail group presenting an average age higher than pre-frail, and the latest higher than non-frail group [9]. Accordingly, we have found a statistically significant difference in age between the three groups, the non-frail individuals being the youngest followed by pre-frail, and frail. Previous studies confirm associations between frailty status and older age [66]; however it should be noted that the chronological age per se cannot justify the development of frailty syndrome, as observed in several studies by the heterogeneity verified within groups with same age [5]. In two Portuguese studies comprising groups of oldest-old adults aged 80 or older (80+) [67] and centenarians [68], authors found representation of the three frailty phenotypes, although mainly of frail subjects (80+: 3.5% non-frail, 24.7% pre-frail, and 71.8% frail; 100+: 5.5% non-frail, 42.9% pre-frail, and 51.6% frail). It is known that the likelihood of developing frailty increases in the oldest-old, above 80 years; nevertheless, non-frail individuals were still found in both studies above these age groups, confirming heterogeneity.

In our study we also found an increased prevalence of women within pre-frail and frail groups. Women usually display greater proportions of frailty when compared to men [66] and several hypotheses are advanced to justify these differences in gender [10]. On one hand, there is the shorter life-expectancy of male individuals compared to women; consequently, the probability of women reaching older ages is greater than men [69]. Since frailty increases with age, this could be an explanation. On the other hand, usually women are more prone to chronic disease burden and disability than men, while males are reported to have an increased frequency of unexpected deaths [70]. Therefore, as well as age, gender is an important confounder when studying frailty syndrome [71].

Polypharmacy is particularly common in older adults and the potential intake of inappropriate medication by this susceptible population is a matter of concern amongst the scientific and clinical community [72]. Our study found a statistically significant association between the increase in the number of drugs consumed and frailty status. This observation is consistent with previous reports [73,74,75]. Many authors suggest that a reduction in polypharmacy may be pivotal to prevent and manage frailty syndrome [76,77]. Furthermore, as recently asserted by Matteo Cesari [78] “the abuse of prescriptions and medical mistakes (usually due to inadequate knowledge of geriatric medicine principles) may dramatically worsen the health status of the aging individual”, consolidating the importance of awareness campaigns for primary care providers regarding polypharmacy in older adults.

Considering the multidimensional nature of frailty syndrome, we also assessed socio-clinical characteristics and geriatric scales on nutrition and dependency. Our study showed that frail subjects were more at risk of malnutrition or malnourished compared to non-frail or pre-frail individuals. These findings are in accordance with previous studies, where nutrition status has been considered a factor closely associated with frailty syndrome [79,80]. Frail subjects also revealed to be dependent on both activities of daily living scales, BADL and IADL. The association between increased dependence and frailty severity is largely reported in other studies [81,82,83]. A systematic review and meta-analysis showed that both pre-frail and frail older adults are more prone to disabilities, as evaluated by BADL and IADL scales [84].

Regarding the influence of frailty syndrome on the biological endpoints studied, our results confirmed an association between frailty and the inflammatory mediators CRP and IL-6. Both endpoints were significantly increased in the presence of frailty. Moreover, CRP levels were 67% and 38% higher in frail and pre-frail subjects, respectively, compared to non-frail. For IL-6 a two-fold increase was observed in frail participants and 50% increase in pre-frail subjects. Several studies have consistently associated frailty syndrome with elevated levels of inflammatory markers, particularly CRP and IL-6 [24,25]. In accordance with our findings, two recent systematic reviews and meta-analyses found strong and significant associations between CRP and IL-6 levels and frailty syndrome in older adults [26,27]. Furthermore, increases in the acute phase protein CRP and the upregulation of pro-inflammatory cytokines such as IL-6 have been associated with increased mortality in older adults [85,86]. IL-6 is produced during the inflammation process and reaches the liver through the bloodstream, where this pro-inflammatory cytokine induces the production of acute phase proteins, as CRP [87]. In line with previous studies supporting an interrelated activation of the inflammatory cascade [24], we did observe that CRP and IL-6 were significantly correlated to each other (*r* = 0.505, *p* < 0.001). Given the known role of IL-6 in CRP regulation, a combination of both endpoints may provide the best prediction of risk associated with systemic inflammation. Furthermore, our results together with the recent published data, indicate that CRP and IL-6 levels may be suitable biological indicators to help identify frailty syndrome in older adults. Additionally, our study shows that IL-6 can distinguish the three frailty phenotypes. Since both markers are currently used in clinical settings, their implementation in primary-care screenings should be fairly simple.

Significant differences were found for all immune activation markers in the presence of frailty, except for Phe and nitrite. However, when adjusting for confounders, significance only remained for neopterin, Kyn/Trp, Phe/Tyr (increased levels), and Trp and Tyr (decreased levels). Only a few studies have evaluated immune activation parameters within the scope of frailty in older adults. Our findings agree with data previously reported by Marcos-Pérez et al. [33]. These authors suggested that the variations observed in the levels of these parameters might be explained by an increased stimulation of the immune system through a more accentuated disturbance of IDO-1 and GTP-CH-I dependent pathways in frail older adults when compared to pre-frail or non-frail individuals. A study with 73 older adults from South Korea suggested Kyn as a potential biomarker of frailty, given the marked increase found in Kyn levels with frailty [34]. In the present study, a significant increase in Kyn levels was also observed in frail subjects when compared to pre-frail or non-frail groups. Nevertheless, when adjusting for confounders, only Kyn/Trp was significantly increased in frail subjects when compared to non-frail.

We found that gender was a modulation factor for some immune endpoints. Males had an 8% and 6% decrease in Tyr and Phe levels, respectively, compared to females, but a 31% increase in nitrite levels. As mentioned, women are generally more burdened with chronic disease than men, and our results are in line with the literature showing that males’ immune systems seem to deteriorate more rapidly and to a greater extent than females [88]. Females have shown stronger innate and adaptive immune responses than males given the faster clearance of pathogens and greater vaccine efficacy [89]. However, this also contributes to their increased susceptibility to inflammatory and autoimmune diseases [89]. These immunological differences between gender are a result of hormonal, genetic, and environmental effects on the immune system [89].

Our study showed significant correlations between CRP and IL-6 and neopterin, Kyn, and Kyn/Trp, supporting the stimulation of the immune system by inflammatory markers. A previous study in older adults also reported Kyn and, Trp-related metabolite levels and Kyn/Trp significantly correlated with IL-6 levels [90].

Interesting results were obtained when the contribution of the five different frailty criteria were independently analysed. Low physical activity, slow walking pace and low grip strength were the criteria that contributed the most, particularly to immune biomarkers. On the other hand, unintentional weight loss had no contribution at all for Kyn/Trp, Tyr levels, and Phe/Tyr alterations. A recent study exploring immune biomarkers in older adults reported associations between Kyn levels and Kyn/Trp and two frailty criteria used—grip strength and gait speed [34]. Nevertheless, a different model was used to assess frailty in this case, the Rockwood Frailty Index. Previous studies using Fried’s Frailty phenotypical criteria had shown a major contribution of the same phenotypical criteria previously pointed out, considering increases in other biomarkers, such as H2AX histone phosphorylation [64] and micronuclei production [91], in a group of older adults. These results can help to improve frailty identification, by approaching a better combination of specific phenotypic criteria and biomarkers.

With respect to vitamin E and oxidative DNA damage, significant differences were found with univariate analysis that did not remain after adjusting for confounders. Although a bidirectional relation between inflammation, induced by immune activation, and chronic oxidative stress has been pointed out to be related to the ageing process [18], there are studies with both positive and negative findings [36,40,41,92]. In a cross-sectional study including 252 Spanish older adults [92] no association was observed between frailty and ROS/RNS (reactive nitrogen species), oxidative DNA damage, and total antioxidant capacity. A study comprising 54 Canadian older adults found no significant differences for vitamins C and E concerning frailty status [41]. Moreover, our results suggest that oxidative stress-related endpoints are not influenced by the presence of frailty. However, data from a larger study (*n* = 827 older adults) reported significant associations between frailty and low vitamin E levels [40]. Hence, the putative association between frailty syndrome and these endpoints is inconclusive. The inconsistency found among studies can be partially explained by the different sample sizes, the different criteria used to assess frailty status, and/or the consideration of confounders in the statistical analysis. Consequently, more research is needed to clarify this controversy.

Together with the notable results obtained regarding inflammation, immune activation, and oxidative stress endpoints with frailty, interesting data on biomarkers predictability to identify this syndrome was also found. The analysis was conducted with the endpoints that presented statistically significant differences among frailty groups, and the best results were obtained for Kyn/Trp, neopterin, and IL-6. Sensitivities between 55 and 83%, and specificities ranging from 60 to 83% were obtained at the optimal predictive values. Thus, levels above 44.70 μmol/mmol of Kyn/Trp, 8.45 nmol/L of neopterin and 4.45 pg/mL of IL-6 can be suggestive of frailty syndrome. However, these results need a careful interpretation, since AUC’s above 0.7 are considered excellent in discriminating the disease status, but lack high accuracy, only obtained at an AUC above 0.9 (outstanding discrimination) [54,55].

In a pilot study, our group reported that consumption of home-produced vegetables was more prevalent in non-frail subjects, as compared to pre-frail individuals (no comparison could be conducted in the frail group). Moreover, decreases in the levels of primary DNA damage were found in participants who consumed home-produced vegetables, within the non-frail and pre-frail groups [46]. Given the previously obtained results, we decided to further explore the influence of diet parameters, such as fruit and vegetables, on some biomarkers studied and their effects on frailty syndrome. The levels of the amino acid Tyr were increased, as well as oxidative DNA damage was decreased, in participants that reported to consume at least 2 portions of fruits and vegetables per day. Additionally, these increases in Tyr concentrations were maintained in the three frailty groups, significant in non-frail and pre-frail individuals. Although no direct relation could be found in the literature between this amino acid and oxidative DNA damage, the role of Tyr kinase on regulatory functions including redox status balance and inflammatory responses, is known [93]. Tyr is involved in the control of several systemic functions, particularly neurotransmitters production and hormones regulation [94,95]. Besides being formed endogenously from Phe, it is also present in food and is commonly used as dietary supplement, namely by patients with Phe-restricted diets [96].

Significant decrease in oxidative DNA damage was just observed within the non-frail group, suggesting that the ingestion of fruit and vegetables is a protective factor in the absence of frailty. Interestingly, previous studies have reported the potential protective factor of fruits and vegetables against frailty [97,98]. García-Esquinas et al. [97] described, in a study in community-dwelling older adults, that consuming three daily portions of fruit or two daily portions of vegetables were strongly associated with lower short-risk of frailty in a dose response manner [97]. A longitudinal study found associations between increases in dietary inflammatory index and higher incidence of frailty, suggesting that a healthier diet could contribute to the prevention of this geriatric syndrome [98]. It was shown that better than supplementation with antioxidant vitamins, a diet rich in natural antioxidants like vitamins E and C, polyphenols and carotenoids positively protects from ROS activity [99]. In fact, studies point out that healthier dietary patterns, such as the Mediterranean diet, rich in fruits and vegetables, can be linked to frailty prevention [100,101].

## 5. Conclusions

In summary, despite the increasing interest of the scientific community on frailty syndrome, there is still a long pathway to get full understanding on how this age-related syndrome may be delayed or prevented, with the ultimate goal of reaching enhanced quality of life for older adults. Nonetheless, a broad knowledge of the multidimensional roots of frailty are recognised in the literature. The causes underlying the loss of physiological reserves are complex and likely related to several factors interacting with one another, such as biological, genetic, psychological, social, and environmental factors.

In our study, we established important associations with biomarkers and some clinical endpoints. Nevertheless, it is important to keep in mind that frailty identification is not standardised yet. The agreement on a gold-standard tool is of paramount importance to make results from different studies comparable and advance frailty research.

Several parameters showed a strong relation with frailty, particularly CRP, IL-6, neopterin, Trp, Kyn/Trp, Tyr, and Phe/Tyr. Our results show that in the presence of frailty there is a progressive immune activation and production of pro-inflammatory molecules, agreeing the with inflammageing model. Therefore, frailty syndrome might also be associated with the progressive impairment of the immune system response resulting in a low chronic pro-inflammatory status. No significant differences or associations, however, were found for oxidative stress parameters; thus, our data does not comply with oxi-inflamm-ageing theory. Several clinical parameters such as nutrition, dependency scales, and polypharmacy were related to frailty and, consequently, may influence the associations observed. Future research should include different dimensions of frailty, including psychological, social, biological, and environmental factors. Finally, it is important to highlight the interesting data of IL-6, neopterin, and Kyn/Trp as potential predictable biomarkers of frailty syndrome in Portuguese older adults.

## Figures and Tables

**Table 1:** Study population.

	Non-Frail	Pre-Frail	Frail	*p*-Value
Total individuals *n* (%)	111 (38.1)	130 (44.7)	50 (17.2)	
Age [years-old] ^a^	73.0 ± 5.9 [65–91]	75.6 ± 6.7 [65–94]	83.6 ± 7.0 [67–94]	**<0.001** ^c^
Gender *n* (%)				**0.003** ^b^
*Males*	59 (53.2)	49 (37.7)	13 (26.0)	
*Females*	52 (46.8)	81 (62.3)	37 (74.0)	
BMI [kg/m^2^] ^a^	24.9 ± 3.6 [22.2–45.0]	28.8 ± 4.2 [20.3–42.2]	28.2 ± 6.3 [16.2–47.9]	0.249 ^c^
Smoking habits *n* (%)				0.569 ^b^
*Non-smokers*	75 (67.6)	95 (73.1)	37 (74.0)	
*Ever smokers*	36 (32.4)	35 (26.9)	13 (26.0)	
*Years smoking ^a^*	25.0 ± 15.8 (0.5–57)	23.7 ± 16.8 (1–67)	26.9 ± 121.0 (2–66)	0.836 ^c^
*Second-hand smokers n (%)*				0.376 ^b^
*No*	65 (58.6)	82 (63.1)	35 (70.0)	
*Yes*	46 (41.4)	48 (36.9)	15 (30.0)	
Alcohol consumption *n* (%)				**<0.001** ^b^
*No*	24 (21.6)	62 (47.7)	27 (54.0)	
*Yes*	87 (78.4)	68 (52.3)	23 (46.0)	
Polypharmacy *n* (%)				**<0.001** ^b^
*No*	77 (69.4)	61 (47.7)	11 (22.0)	
*Yes*	34 (30.6)	67 (52.3)	39 (78.0)	
*No. drugs consumed/day ^a^*	3.6 ± 2.5 (0–13)	5.1 ± 2.6 (1–13)	7.2 ± 3.4 (1–15)	**<0.001** ^c^
Vitamin supplementation *n* (%)				0.224 ^b^
*No*	109 (98.2)	122 (93.8)	47 (94.0)	
*Yes*	2 (1.8)	8 (6.2)	3 (6.0)	
Vaccination in the last year *n* (%)				0.104 ^b^
*No*	30 (27.0)	22 (16.9)	8 (16.0)	
*Yes*	81 (73.0)	108 (83.1)	42 (84.0)	
Nutrition *n (%)*				**<0.001** ^b^
*Normal nutrition status*	110 (99.1)	115 (88.5)	24 (48.0)	
*At risk or malnourished*	1 (0.9)	15 (11.5)	26 (52.0)	
Functional status-BADL *n* (%)				**<0.001** ^b^
*No dependence*	98 (88.3)	91 (70.0)	13 (26.0)	
*Dependence*	13 (11.7)	39 (30.0)	37 (74.0)	
Functional status-IADL *n* (%)				**<0.001** ^b^
*No dependence*	102 (91.9)	96 (73.8)	5 (10.0)	
*Dependence*	9 (8.1)	34 (26.2)	45 (90.0)	
Falls in the last 6 months ^a^ (self-reported)	0.3 ± 1.2 (0–12)	0.4 ± 0.9 (0–6)	0.4 ± 0.7 (0–3)	0.714 ^c^

^a^ mean ± standard deviation (range); ^b^ Chi-square test (bilateral); ^c^ ANOVA test (bilateral). BADL: basic activities of daily living; BMI: body mass index; IADL: instrumental activities of daily living.

**Table 2:** Results of biomarkers in the study group, classified according to frailty status (univariate analysis).

	Non-Frail				Pre-Frail				Frail				*p*-Value ^#^
	*n*	Mean		SE	*n*	Mean		SE	*n*	Mean		SE	
CRP (mg/dL)	109	0.24	±	0.02 ^a^	130	0.61	±	0.13 ^b^	49	0.72	±	0.16 ^b^	**0.002**
IL-6 (pg/mL)	109	2.18	±	0.17 ^a^	130	4.10	±	0.54 ^b^	50	6.42	±	1.04 ^c^	**<0.001**
Neopterin (nmol/L)	106	8.19	±	0.44 ^a^	128	10.27	±	0.51 ^b^	48	14.26	±	1.58 ^c^	**<0.001**
Tryptophan (μmol/L)	106	62.99	±	1.06 ^a^	128	60.31	±	1.11 ^a^	48	53.92	±	1.85 ^b^	**<0.001**
Kynurenine (μmol/L)	106	2.33	±	0.05 ^a^	128	2.47	±	0.06 ^a^	48	2.87	±	0.12 ^b^	**<0.001**
Kyn/Trp (μmol/mmol)	106	37.94	±	1.14 ^a^	128	42.28	±	1.18 ^b^	48	56.20	±	3.06 ^c^	**<0.001**
Tyrosine (μmol/L)	106	85.36	±	1.99 ^a^	128	79.99	±	2.03 ^a^	48	75.16	±	2.93 ^b^	**0.018**
Phenylalanine (μmol/L)	106	89.89	±	1.75 ^a^	128	89.10	±	1.64 ^a^	48	85.68	±	2.86 ^a^	0.413
Phe/Tyr (μmol/μmol)	106	1.08	±	0.02 ^a^	128	1.17	±	0.02 ^b^	49	1.17	±	0.03 ^a,b^	**0.012**
Nitrite (μmol/L)	102	26.33	±	1.96 ^a^	125	24.15	±	1.68 ^a^	39	26.83	±	3.29 ^a^	0.783
Vitamin A (mg/L)	109	0.52	±	0.01 ^a^	129	0.49	±	0.01 ^a^	49	0.50	±	0.03 ^a^	0.323
Vitamin E (mg/L)	109	14.11	±	0.27 ^a^	129	13.51	±	0.26 ^a,b^	49	12.86	±	0.48 ^b^	**0.046**
Net FPG-sensitive sites (%tDNA)	107	9.37	±	0.47 ^a,b^	127	10.15	±	0.49 ^a^	48	8.11	±	0.71 ^b^	**0.024**

^#^ Multiple group comparison (ANOVA). Different letters indicate statistically different groups (Tukey’s test).

**Table 3:** Effect of fruit/vegetables consumption on tyrosine and oxidative DNA damage; models adjusted by age, gender, smoking habits, and actual confounders.

	Tyrosine		Net FPG-Sensitive Sites	
	Mean Ratio	95% CI	Mean Ratio	95% CI
Fruits and vegetables consumption >2/day				
*No consumption*	1.00		1.00	
*Consumption*	1.15 *	(1.02–1.29)	0.69 **	(0.53–0.89)

CI: confidence interval; * *p* < 0.05; ** *p* < 0.01.

## Data Availability

The data presented in this study are available in this article.
